# Supplementary material for: Splice-Junction-Based Mapping of Alternative Isoforms in the Human Proteome
Source: Cell Rep. Author manuscript; Available in PMC 2020 Jan 15. (PMC6961840; doi:10.1016/j.celrep.2019.11.026)
Supplement: 2 [file NIHMS1546469-supplement-2.zip › DF1/PXD010154/Liver/Liver_37_ALDH1L1_GAASSVLELTEAELVTAEAVRK.pdf]

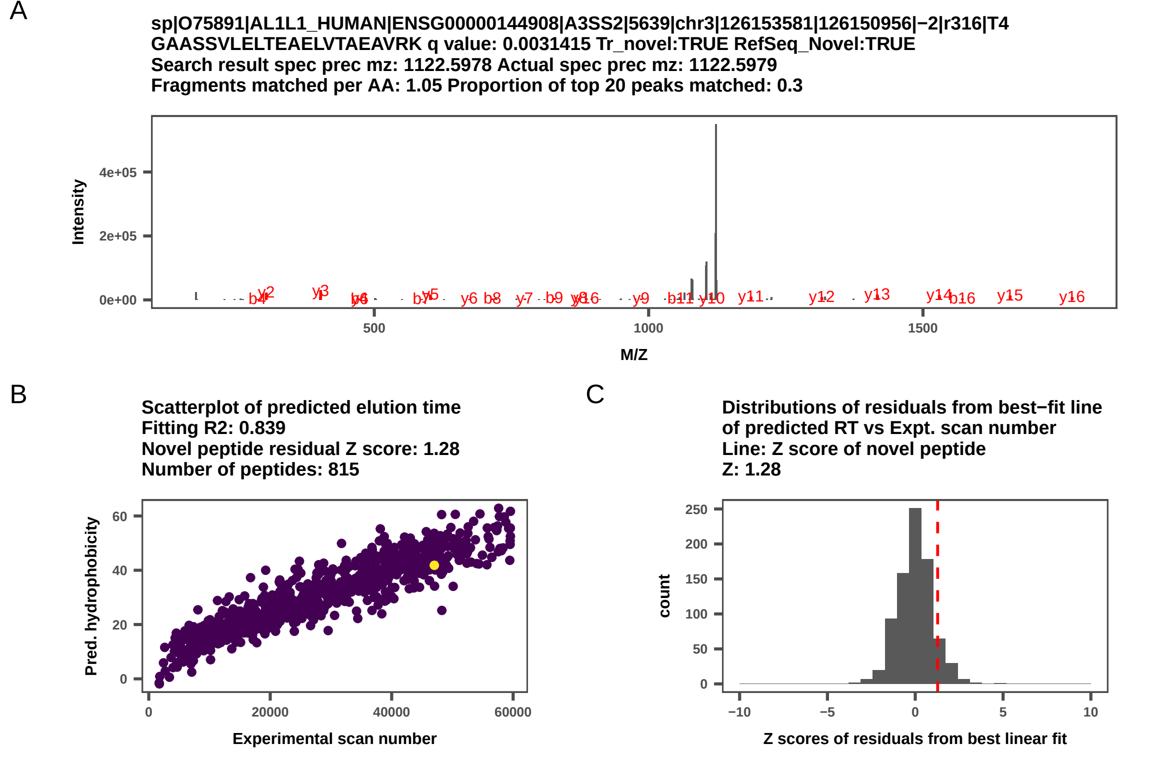
A
sp|O75891|AL1L1_HUMAN|ENSG00000144908|A3SS2|5639|chr3|126153581|126150956|−2|r316|T4
GAASSVLELTEAELVTAEAVRK q value: 0.0031415 Tr_novel:TRUE RefSeq_Novel:TRUE
Search result spec prec mz: 1122.5978 Actual spec prec mz: 1122.5979
Fragments matched per AA: 1.05 Proportion of top 20 peaks matched: 0.3
Intensity
4e+05
2e+05
0e+00
b4
y2
y3
b6
y5
b7
y6
b8
y7
b9
y8
b16
y9
b11
y10
y11
y12
y13
y14
b16
y15
y16
500
1000
1500
M/Z
B
Scatterplot of predicted elution time
Fitting R2: 0.839
Novel peptide residual Z score: 1.28
Number of peptides: 815
Pred. hydrophobicity
60
40
20
0
0
20000
40000
60000
Experimental scan number
C
Distributions of residuals from best−fit line
of predicted RT vs Expt. scan number
Line: Z score of novel peptide
Z: 1.28
count
250
200
150
100
50
0
−10
−5
0
5
10
Z scores of residuals from best linear fit
